# Simultaneous screening of overexpressed genes in breast cancer for oncogenic drivers and tumor dependencies

**DOI:** 10.1038/s41598-024-64297-w

**Published:** 2024-06-09

**Authors:** Adaobi Mofunanya, Eleanor R. Cameron, Christian J. Braun, Frank Celeste, Xiaoyu Zhao, Michael T. Hemann, Kenneth L. Scott, Jinyu Li, Scott Powers

**Affiliations:** 1 Department of Pathology, Stony Brook Cancer Center, Stony Brook, NY 11794 USA; 2 Koch Institute for Integrative Cancer Research and Department of Biology, Massachusetts Institute of Technology, Cambridge, MA 02139 USA; 3 Graduate Program in Genetics, Stony Brook University, Stony Brook, NY 11794 USA; 4 Graduate Program in Molecular and Cellular Biology, Stony Brook University, Stony Brook, NY 11794 USA; 5 Department of Molecular and Human Genetics, Baylor College of Medicine, Houston, TX 77030 USA

**Keywords:** Cancer genomics, Cancer, Genetics

## Abstract

There are hundreds of genes typically overexpressed in breast cancer cells and it's often assumed that their overexpression contributes to cancer progression. However, the precise proportion of these overexpressed genes contributing to tumorigenicity remains unclear. To address this gap, we undertook a comprehensive screening of a diverse set of seventy-two genes overexpressed in breast cancer. This systematic screening evaluated their potential for inducing malignant transformation and, concurrently, assessed their impact on breast cancer cell proliferation and viability. Select genes including *ALDH3B1*, *CEACAM5*, *IL8*, *PYGO2*, and *WWTR1*, exhibited pronounced activity in promoting tumor formation and establishing gene dependencies critical for tumorigenicity. Subsequent investigations revealed that *CEACAM5* overexpression triggered the activation of signaling pathways involving β-catenin, Cdk4, and mTOR. Additionally, it conferred a growth advantage independent of exogenous insulin in defined medium and facilitated spheroid expansion by inducing multiple layers of epithelial cells while preserving a hollow lumen. Furthermore, the silencing of CEACAM5 expression synergized with tamoxifen-induced growth inhibition in breast cancer cells. These findings underscore the potential of screening overexpressed genes for both oncogenic drivers and tumor dependencies to expand the repertoire of therapeutic targets for breast cancer treatment.

## Introduction

Genome-wide estimates of the number of genes overexpressed in specific cancer types range in the low hundreds to high hundreds^1,2^. Depending upon their expression pattern in cancers, these genes can be useful biomarkers for detection, prognosis, or prediction of treatment response. However, determining whether they promote tumorigenicity or evaluating their utility as potential therapeutic targets requires functional analysis. Overexpressed genes may be growth-inhibitory, particularly if their overexpression results from a dysregulated negative feedback loop. For example, the HPV oncoprotein E7 binds and induces proteolytic degradation of the Rb protein, which relieves the tumor suppressor gene p16^Ink4a^ from negative feedback control, leading to its upregulation^3,4^. Likewise, *BRAF-V600E* mutant melanomas and *KRAS*-mutant lung adenocarcinomas both overexpress several inhibitors of the mitogen-activated kinase pathway^5,6^. In these cases, the primary genetic alteration blocks the negative feedback exerted by the overexpressed inhibitory genes.

Single-gene, small-scale functional analysis of overexpressed genes has led to the generation of hundreds of proposed tumor-promoting genes^7^. At present, it is difficult to evaluate many of these studies since they often use unique experimental conditions to determine oncogenicity. More systematic approaches that survey the induced oncogenic phenotypes of multiple candidate genes have the advantage of providing several internal comparisons. Using cDNA-based or open-reading-frame (ORF) overexpression, systematic screening has identified oncogenic roles for *PVRL4* in breast cancer and proliferation of T-cells by *LTBR*^8,9^. In addition to these systematic screens for genes capable of promoting the tumorigenicity or proliferation, overexpression screens have also been used to identify *GATAD2B* and *PYGO2* as drivers of metastasis^9,10^ as well as the chromatin-modifying complex SAGA as a driver of pluripotency^11^.

In this study, we set out to determine what percentage of overexpressed genes in breast cancer could promote tumorigenicity of premalignant mammary epithelial cells. We performed pooled screening with barcoded ORFs and measured the relative number of mammary epithelial cells expressing each individual ORF both before and after growth in three different conditions, including in vivo tumor formation. In parallel, we used shRNA screening to determine if the overexpression of these genes was selectively required in breast cancer cell lines. To our knowledge, this is the first report on concurrent screening for drivers and dependencies. Additionally, we used the proteomic method of reverse phase protein array (RPPA) profiling of protein levels and phosphorylation to gain valuable insights into the cancer biology and oncogenic mechanisms of our lead candidates.

## Results

### Selection of overexpressed genes and transfection of LentiORFs into both MCF10A and NMuMG

We selected genes based on their overexpression in breast cancer and their presence in a previously acquired Precision LentiORF expression library containing 3526 ORFs. We applied both outlier and cancer vs. normal differential analysis of breast cancer TCGA data using Oncomine Research tools and chose the top 100 overexpressed genes for functional studies (Supplementary Table 1). We performed a pilot experiment with ten of the chosen ORFs and measured the ability of amplicon sequencing to detect the different ORFs after transfection into MCF10A cells, plasmid integration and selection, and one week of growth before amplifying the ORFs from genomic DNA using universal 5′ and 3′ ORF primers. Based on the uneven amplification of the ORFs, we decided to switch to a 24-bp barcoded ORF system which promised to yield more even amplification of the uniform-length and balanced nucleotide composition barcodes. We tagged each individual ORF with a unique barcode for next-generation sequencing and inserted the tagged-ORF into the pLenti6.3 R1R2 lentiviral expression vector using the production pipeline previously described^12^. We successfully transferred and sequence-verified seventy-two ORFs, and these barcoded-ORF expression vectors were transfected individually (to ensure an initial even representation) into the immortalized human mammary epithelial cell line MCF10A. MCF10A resemble primary breast epithelial cells in that they require substrate attachment, growth factors and hormones for growth, are contact-inhibited, and are not tumorigenic. They have two cancer-related genetic alterations: homozygous deletion of the *CDKN2A* locus and amplification of *MYC*, and this allows them to be transformed in vitro by mutant *PIK3CA* or *RAS* oncogenes, but acquiring the ability to form carcinomas in vivo requires several additional oncogenic alterations^13^. Mouse cells require fewer genetic alterations to form tumors, thus for in vivo screening we chose the normal murine mammary epithelial cell line NMuMG, which is heterozygous for a missense *TP53* mutant (R277C) and forms tumors when transfected with powerful oncogenes such as mutationally activated *RAS*^14^. In order to allow NMuMG cells to form tumors with weaker oncogenes, we overexpressed *MYC*, a procedure that we had used previously in our cDNA screen for tumor-promoting genes using *TP53* mutant hepatoblasts^15^. As we did with MCF10A, the seventy-two barcoded-ORF expression vectors were transfected individually into NMuMG-Myc cells.

Following selection of transfected cells with blasticidin, the individual transfectants were cultured briefly and then equal cell numbers of each were combined into a pool. Each pool was then divided into three separate cultures to generate biological replicates. After three days, there were sufficient cells from each replicate to use for isolation of genomic DNA, PCR amplification of barcodes, and amplicon sequencing to determine the baseline representation of the different ORF-transfectants. The MCF10A ORF-transfectant pool replicates were then placed into 3D culture (Matrigel) for three weeks to allow for spheroid formation, and in parallel grown for three weeks under standard 2D cell culture conditions (with passaging as needed to avoid confluence). Each of the NMuMG-Myc ORF-transfectant pool replicates were injected subcutaneously into immunodeficient mice and the mice were sacrificed after eight weeks and the tumors removed. None of the mice injected with control NMuMG-Myc empty vector transfectants formed tumors after eight weeks.

For each of these growth conditions, genomic DNA was isolated and the barcode read counts determined by amplicon sequencing. Following preprocessing, normalization, and log-transformation of the barcode read counts, the ORF frequencies in the three biological replicates of the initial time point were compared to the ORF frequencies in the latter time points.

### Overexpressed genes vary widely in their effects on growth and tumor formation

**Figure 1:**
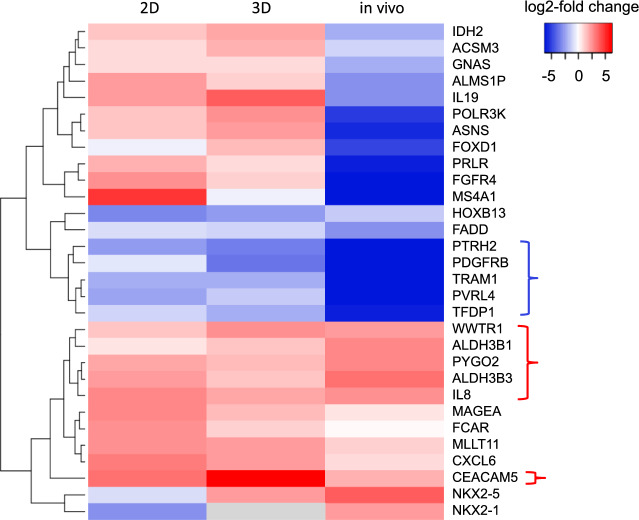
Heatmap visualization and hierarchical clustering of the growth phenotypes induced by overexpression of different ORFs. ORF library transfectants of MCF10A were grown in standard cell culture (2D) or suspension in matrigel (3D), and ORF library transfectants of NMuMG were screened in vivo. Log2-transformed changes in ORF proportions compared to initial library transfectants were determined by barcode-sequencing. Significance of log2-transformed changes were assessed using a Student's t-test (see text). The red brackets signify ORFs that showed enrichment in all three assays, whereas the blue bracket signifies the opposite.

The values and statistical tests of log2-fold changes in the seventy-two ORF proportions for the three growth and/or tumor assays is tabulated in Supplementary Table 2. Filtering for statistical significance (p < 0.05) reduced the number of ORFs to thirty, and we used a heatmap with hierarchical clustering to examine the overall patterns of log2-fold enrichment or depletion. One set of genes that clustered together showed enrichment in all three assays, and included *ALDH3B1*, *ALDH3B2*, *IL8*, *PYGO2*, and *WWTR1* (Fig. 1). *CEACAM5* also showed enrichment in all three assays, even though it was a member of a distinct but nearby cluster (Fig. 1). Interestingly, *CEACAM5* was also the strongest inducer of growth in 3D spheroids (Supplementary Table 2).

Several of these genes have been previously shown to either promote tumorigenicity or tumorigenic properties. These include the proinflammatory cytokine *IL8* which stimulates the growth of triple-negative breast cancer cells^16^; the transcriptional activator *WWTR1* which induces cancer stem-cell phenotypes in breast cancer^17^; and *CEACAM5* and *PYGO2* which have both been shown to promote metastasis when overexpressed^9,18^. Although not proven to be oncogenic, *ALDH3B1* and *ALDH3B2* are members of the aldehyde dehydrogenases family that are highly expressed in therapy-resistant cells in different cancer types^19^.

At the other end of the spectrum in Fig. 1, there is a cluster of genes with very high tumor-inhibitory activity which includes *PDGFRB*, *TRAM1*, *PVRL4*, *TFDP1*, and *PTRH2* (Fig. 1). Surprisingly, three of these genes have been shown to promote oncogenic properties when overexpressed^20–23^. Although our results with these genes appear to be contradictory, we note that extensively validated cancer genes (*RUNX1* and *NOTCH* family members) have been shown to have dual roles of either tumor suppression or survival/tumor promotion depending upon the specific cell type^24,25^. We think it is likely that some overexpressed genes will also have opposing roles that are cell-type dependent.

### Tumor dependencies revealed by shRNA screening

**Figure 2:**
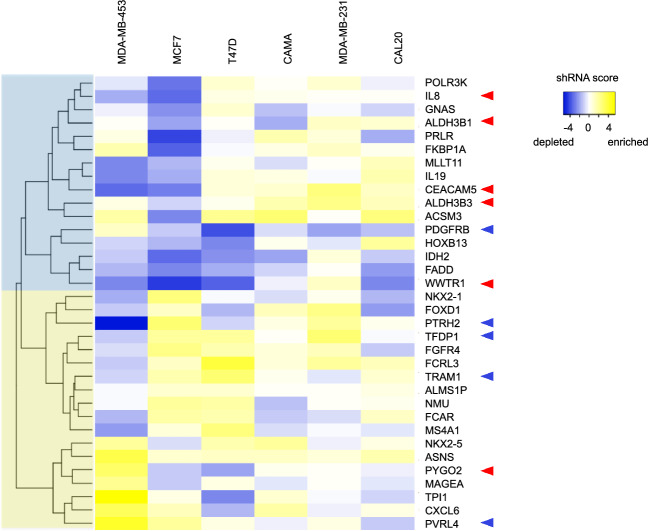
Heatmap visualization and hierarchical clustering of gene inhibition mediated by shRNAs in a panel of five breast cancer cell lines. shRNA library transfectants of the five breast cancer cell lines were grown in standard cell culture for two weeks. Log2-transformed changes in shRNA proportions compared to initial library transfectants were determined by barcode-sequencing. Data was transformed into Z-scores for data visualization. Blue shading indicates slightly higher growth inhibition by shRNAs, whereas yellow shading indicates the opposite. Red arrows point to genes that promoted growth in all three growth assays per Fig. 1, blue arrows indicate genes that inhibited growth in all three assays.

In parallel, we examined the tumor dependencies in five different breast cancer cell lines by pooled shRNA screening of the same set of seventy-two overexpressed genes. Focusing on the subset of thirty genes with significant results in the ORF screening, we used a heatmap with hierarchical clustering to examine the overall patterns of shRNA enrichment or depletion. Although there was pronounced variability between the cell lines, we detected two gene clusters, one showing slightly higher growth inhibition by shRNAs (shRNA depletion), and another showing slightly less growth inhibition (shRNA enrichment) (Fig. 2). Five out of six oncogenic genes from the ORF screen were in the cluster of genes showing slightly higher growth inhibition by shRNAs, and four out of five inhibitory genes from the ORF screen were in the other cluster of genes showing slightly less growth inhibition (Fig. 2). These results suggest that there is an association of growth and tumor-promoting ability with the capability of targeting shRNAs to inhibit growth and/or survival.

### Correlation between tumor promoting ability and tumor dependency

To quantitatively assess the relationship between growth and tumor-promoting ability and shRNA growth inhibition, we created a series of summary statistics for shRNA depletion for all five cell lines, including the minimum value, first quartile, and mean. The most significant correlation was between the first quartile summary shRNA statistic and tumor-forming ability. To switch to the more intuitive concept of tumor dependency, we used the inverse of the shRNA inhibition summary statistic. Plotting tumor-forming ability versus tumor dependency revealed a weak positive correlation (*r* = 0.24) which was not significant based on the standard cutoff of 0.05 (p < 0.108), yet the trend is clear (Fig. 3).

**Figure 3:**
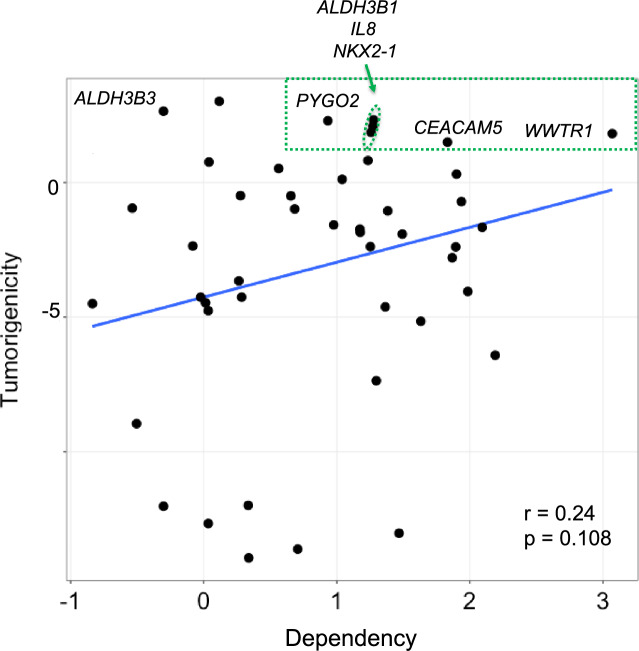
Relationship between induction of tumorigenicity and tumor dependency. Scatter plot of the tumor dependency scores (see text) on the x-axis, and tumorigenicity scores on the y-axis. The indicated genes correspond to ORFs that showed enrichment in all three assays (see Fig. 1) except for *NKX2-1* (see text). The fitted line represents the linear relationship between the two variables, which did not pass the significance threshold of p < 0.05 but clearly indicated a trend.

The upper right section of the graph in Fig. 3 shows genes with concomitant high tumor-promoting activity and high tumor dependency and includes five of the six genes from the growth and tumor-promoting cluster (see Fig. 1). Also within this group is a tumor-promoting gene (*NKX2-1*) was not in the growth and tumor-promoting cluster because it failed to promote growth in cell culture (see Fig. 1)*.* Conversely, a single gene from the growth and tumor-promoting cluster, *ALDH3B2,* showed low tumor dependency scores (Fig. 3). Whether these results indicate that concurrent screening for drivers and dependencies are an effective way to identify potential therapeutic targets is addressed in the discussion section.

### Confirmation that *CEACAM5* overexpression induces tumorigenicity

As pointed out earlier, the cell adhesion gene *CEACAM5* was the strongest inducer of growth in 3D spheroids—more than threefold greater than the next strongest inducer of growth in 3D spheroids (*IL19*) (Fig. 1, Supplementary Table 2). These findings suggest that the oncogenic mechanism of *CEACAM5* differs from that of other drivers. Based on this, we focused our follow-up studies on *CEACAM5*. We first tested the validity of the pooled screening result that *CEACAM5* overexpression promoted the tumorigenicity of NMuMG-Myc cells. NMuMG-Myc cells were transduced with a lentiORF *CEACAM5* expression construct and shown by quantitative RT-PCR to express high levels of *CEACAM5*. We determined that *CEACAM5* overexpression induced rapidly growing tumors following subcutaneous injection of 5 × 10^6^ cells. Over the same period of four weeks, orthotopic injection of 5 × 10^6^ cells into mammary fat pads produced slightly smaller tumors. Control NMuMG-Myc cells did not form tumors under either of these conditions (Supplementary Fig. 1).

### shRNA mediated suppression of *CEACAM5* in reduces clonogenic growth in breast cancer cell lines and enhances tamoxifen-growth inhibition

To validate the pooled shRNA screening results indicating that some breast cancer cell lines are dependent on expression of *CEACAM5* for growth, we constructed two mir-30 based shRNAs plasmids targeting *CEACAM5* based on the two strongest shRNAs from the pooled screening results. Following lentiviral transfection, we determined that each one had strong on-target activity in suppressing *CEACAM5* expression (Fig. 4A). Both shRNAs suppressed the clonogenic growth of two breast cancer cell lines, T47D and MCF7, but neither shRNA was able to suppress the clonogenic growth of unrelated kidney epithelial 293 T cells (Fig. 4B).

**Figure 3:**
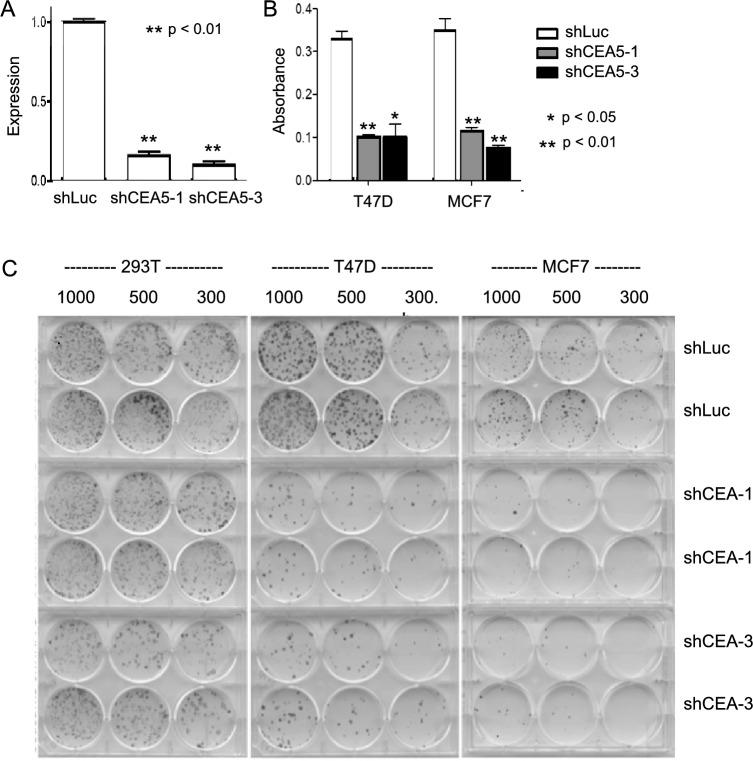
shRNA silencing of *CEACAM5* inhibits the clonogenic growth of breast cancer cell lines. Panel (**A**) Validation of on-target effects of shRNAs targeting *CEACAM5*. T47D cells were transfected with either a shRNA targeting luciferase (control) or two independent shRNAs targeting *CEACAM5* (Ceacam5-1 and Ceacam5-3). Following selection for stable transfectants, quantitative RT-PCR analysis of *CEACAM5 RNA* was performed for expression levels of on RNA extracted from transfected T47D cells. GAPDH expression was used for normalization and results were represented as expression relative to T47D-luc. Panel (**B**) Quantification of clonal growth over 3 weeks in cell lines infected with shRNAs against *CEACAM5* (Ceacam5-1 and Ceacam5-3) relative to a shRNA against luciferase (control). Panel (**C**) Colony formation assay of 293 T, T47D and MCF7 cells infected with shRNA against luciferase (control) or *CEACAM5* (Ceacam5-1 and Ceacam5-3). The significance of the findings was assessed using a Student's t-test, while error bars depict the standard error of the means.

**Figure 5:**
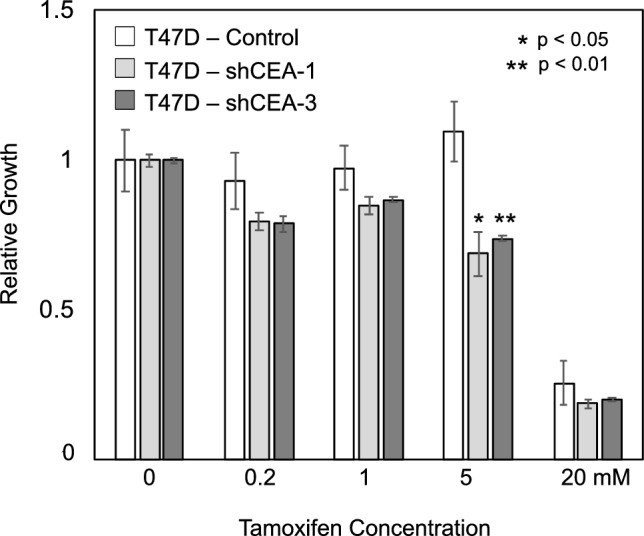
Silencing of *CEACAM5* sensitizes T47 breast cancer cell line to growth inhibition by tamoxifen. Quantification of viability of T47D cells and T47D-*CEACAM5* knockdown derivatives after four days growth in standard medium with the indicated concentrations of tamoxifen.

We noted that high *CEACAM5* protein expression as ascertained by immunohistochemistry has been associated with resistance to endocrine therapy in breast cancer patients^26^. Based on this, we examined whether suppression of *CEACAM5* expression could modify growth-inhibitory effects of tamoxifen in the two ER-positive cell lines T47D and MCF7. Although suppression of *CEACAM5* expression did not modify the response of MCF7 (data not shown), it enhanced growth inhibition by tamoxifen in T47D (Fig. 5). This result suggests that it is possible that inhibition of *CEACAM5* could be of therapeutic benefit in treating breast cancer patients.

### Overexpression of *CEACAM5* obviates the need for insulin in defined medium and promotes spheroid growth by a unique mechanism

**Figure 6:**
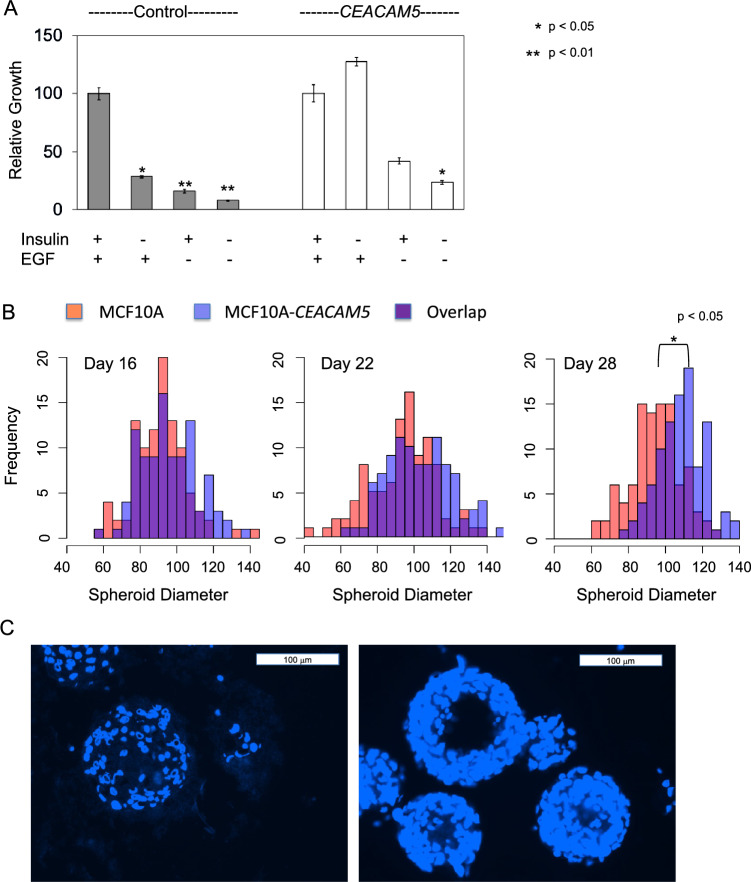
Effects of *CEACAM5* on growth-factor requirements and on 3D spheroid growth. Panel (**A**) Growth of control MCF10A compared to the *CEACAM5*-MCF10 in minimal medium with or without added growth factors (EGF, insulin). All values normalized were to growth with both EGF and insulin. The significance of the findings was assessed using a Student's t-test, while error bars depict the standard error of the means. Panel (**B**) Effect of *CEACMA5* overexpression on 3D growth in matrigel. MCF10a (red shading) and *CEACAM5*-MCF10A (blue shading) were cultured on matrigel. Acini structures were photographed on days indicated and their diameters were assessed using Leica Q500 MC Qwin software. The significance of the difference in the means of the diameters was assessed using a Student's t-test. Panel (**C**) Acini structures from MCF10a and MCF10a-CEACAM5 grown in matrigel were fixed, embedded, and stained with DAPI.

We tested the ability of *CEACAM5* overexpression to substitute for specific growth factors for cell culture growth. *CEACAM5* overexpressing cells were completely independent of insulin as a growth factor, and although less dependent upon EGF than control MCF10A cells, they still showed EGF dependence (Fig. 6A). To validate the ORF screening results that indicated that overexpression of *CEACAM5* greatly increased the growth of MCF10A cells in 3D culture, we examined MCF10A-*CEACAM5* after growth in a 3D matrigel matrix and determined that *CEACAM5* overexpression induced a modest but significant increase in the average diameter of spheroids formed in matrigel, as visualized by a time-series of frequency distributions of spheroid sizes (Fig. 6B). This modest increase could not explain the stronger growth predicted from the screening results. However, examination of spheroids after they were fixed, sectioned, and visualized by staining with DAPI revealed that *CEACAM5* overexpression increased growth significantly within the spheroid itself. Unlike the oncogenes *ERBB2*, *PIK3CA,* or *WWTR1* previously tested in the MCF10A spheroid formation assay, *CEACAM5* overexpression did not alter the spheroid morphology or prevent hollowing out of the inner mass of cells^27^, but instead induced a notable increase in growth in the spheroid periphery, increasing the number of epithelial cell layers (Fig. 6C).

### RPPA analysis of the effects of *CEACAM5* overexpression on the functional proteome

**Figure 7:**
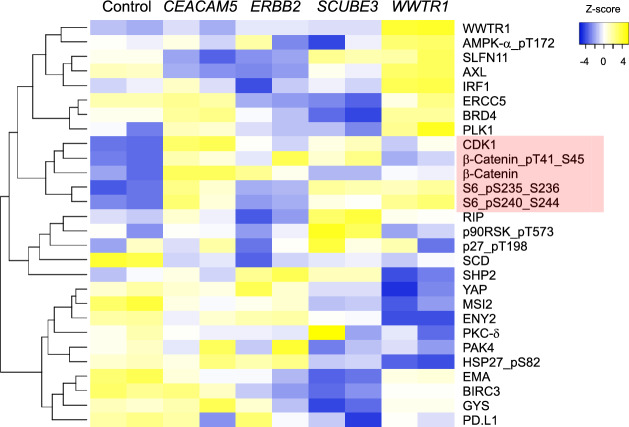
Oncogenes induce diverse alterations of protein levels and protein phosphorylation. Heatmap and hierarchical clustering of changes in log2-fold protein phosphorylation and total protein levels in different oncogene transformants of MCF10. Normalization was based on the 408 protein measurements made with each sample. The red shading highlights a cluster of proteins whose total levels or phosphorylation are higher in *CEACAM5*-transformants than they are in the other three transformants (CDK1, β-Catenin, phosphorylated β-Catenin) or tied for highest with *WWTR1*-transformants (two different phosphorylation sites for ribosomal protein S6).

To gain insight into the biochemical mechanisms by which *CEACAM5* overexpression induces tumorigenicity, we used the high-throughput reverse-phase protein analysis (RPPA) pipeline established at MD Anderson to examine how *CEACAM5* expression affected levels and post-translational modifications of several key proteins involved in growth control and other cancer-related phenotypes^28^. We transfected MCF10A cells with either the *CEACAM5* ORF expression construct, or *WWTR1-, ERBB2-, SCUBE3-*ORF expression constructs for comparison, along with empty vector as a control. Following selection for stable integration, we cultured the cells briefly in standard medium and prepared biological triplicates for RPPA analysis. Out of a total of 304 protein measurements, 93 were significantly different between the five groups (ANOVA p < 0.01). To visualize differences more readily between the five groups, we used a more stringent significance cut-off (p < 0.001) which reduced the number to 28. We used a heatmap with hierarchical clustering to examine the overall patterns of protein enrichment or depletion (Fig. 7). The bottom 10 proteins in the heatmap are expressed relatively higher in normal, empty-vector MCF10A cells, whereas the top 18 proteins are expressed in relatively lower levels in control MCF10A cells (Fig. 7). Within this top cluster is the middle branch of 5 proteins that are preferentially expressed in MCF10A-*CEACAM5* cells, most notably β-catenin (both total and phosphorylated), indicating strong activation of the canonical Wnt-signaling pathway (Fig. 7). The possibility that this underlies the mechanism by which MCF10A-*CEACAM5* spheroids form multiple epithelial cell layers is addressed in the discussion. Also included in this group is CDK1, which in addition to its role in cell cycle progression also contributes to cancer progression^29^. Also notable is the activation of mTOR as indicated by increased phosphorylation of its canonical substrate, ribosomal subunit S6, a property it shares with WWTR1 (Fig. 7).

**Figure 8:**
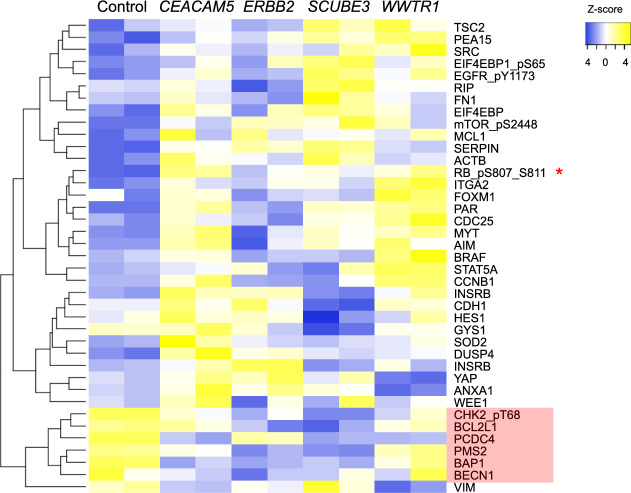
Overexpression of *CEACAM5* induces phosphorylation of retinoblastoma protein and lowers protein levels of tumor suppressors. Heatmap and hierarchical clustering of relative changes in log2-fold protein phosphorylation and total protein levels in different oncogene transformants of MCF10. Normalization was based on the 408 protein measurements made with each sample. A red asterisk marks phosphorylated retinoblastoma protein (RB_pS807_S811). The red shading indicates a cluster of tumor suppressor proteins whose levels are lower in some or all the oncogene transformants.

We reasoned that some of the proteins that were significantly altered at the p < 0.01 level but not at the p < 0.001 level might be relevant to how *CEACAM5* transforms cells. We did not analyze any protein that was not significantly different between control MCF10A and *CEACAM5*-MCF10 (t-test p < 0.05). We detected increased phosphorylation of the RB1 protein, indicating activation of CDK4/6 signaling (Fig. 8). Additionally, we found four proteins with tumor suppressive properties that clustered together and were significantly reduced in *CEACAM5*-MCF10, including BECN, BAP1, PCDC4, and PMS2 (Fig. 8). The modest suppression of BCL2L1/Bim by overexpression of *CEACAM5* is consistent with the formation of empty spheroid lumen as discussed below.

## Discussion

The primary motivation behind this investigation was to ascertain the proportion of overexpressed genes in breast cancer that might function as oncogenic drivers and serve as potential tumor dependencies and therapeutic targets. Our analysis revealed that six out of the seventy-five genes scrutinized met these criteria. This observation, considering the hundreds of genes overexpressed in breast cancer, implies the existence of a greater number of therapeutic targets than previously envisaged. However, it is noteworthy that five of the identified genes were previously shown to possess oncogenic properties in breast cancer, with *ALDH3B1* being the sole exception. The quest for genuinely novel targets may necessitate pooled screening conducted at a significantly larger scale.

Conducting pooled screens presents notable technical challenges, irrespective of whether involving ORFs, shRNAs, or CRISPR technology. Rigorous quality control measures aimed at minimizing technical variability between biological replicates, genomic DNA purifications, and the generation of amplicons for DNA sequencing demand a high level of technical proficiency. Scaling such endeavors to encompass a broad scope surpasses the capabilities of individual laboratories. Although larger institutions have managed to scale screening efforts across numerous cell lines employing genome-wide shRNA or CRISPR/Cas9 libraries^30,31^, these initiatives have been confined to standard 2D cell culture conditions. Furthermore, parallel screening with ORFs has not been undertaken, and thus far, targets identified have not culminated in the development of new cancer therapeutics. It is our contention that concurrent screening for drivers and dependencies, particularly if incorporating in vivo assays, would be more effective at uncovering authentic cancer therapeutic targets.

A weakness of this study was not performing all three growth or tumorigenicity assays using the same cell line. MCF10A is an immortalized human mammary epithelial cell line with a homozygous deletion of the *CDK2NA* locus. NMuMG is an immortalized murine mammary epithelial cell line harboring a *Trp53* mutation. These two different genetic alterations both inactivate the p53 pathway, so the genetic contexts are similar. But the species are different. Our rationale for choosing MCF10A is that it is the most thoroughly studied normal mammary epithelial cell line, and being human it offered the potential of profiling of protein phosphorylation and protein levels by RPPA. However, normal human cells require six or more genetic alterations to become tumorigenic whereas their murine counterparts only require two^32^. From a practical standpoint, we chose to screen for tumor formation using NMuMG.

Another weakness of this study is the possibility that a given human gene cannot functionally substitute for their murine counterpart. This might explain why some genes promoted growth in MCF10A but not tumor formation in NMuMG, such as IL19 which promoted tumor formation in a previous study^33^.

The impetus for choosing *CEACAM5* for further study was our observation that it was the most potent gene at inducing growth of 3D spheroids. Additionally, although a previous report showed that *CEACAM5* overexpression tumor outgrowth at metastatic sites^18^, it did not show that *CEACAM5* overexpression could induce primary tumor growth and other oncogenic properties associated with pre-metastatic cancer. Our identification of an oncogenic role for *CEACAM5* in primary tumor growth aligns with its histological presence in early breast cancer stages^34^. Also aligning with a role in primary tumor growth is our finding that overexpression allowed for growth in minimal medium without added insulin.

We also found *CEACAM5* overexpression increased 3D spheroid growth of MCF10A cells by forming multiple layers of epithelial cells, while still forming a hollow lumen as normal MCF10A. The combination of these two effects are similar to the hyperproliferation of epithelial cells seen in some early precursor lesions of breast cancer^35^. A similar effect was observed in the colon of transgenic mice with multiple copies of the human locus containing *CEACAM5* and its close homolog *CEACAM6.* In addition to hyperproliferation, the colon of these mice showed increased crypt fission, a process that increases the number of epithelial cell layers^36^. By using RPPA proteomic profiling, we found that *CEACAM5* overexpression activated β-catenin signaling. Activation of β-catenin- signaling may be the mechanism by which *CEACAM5* overexpression causes multiple layers of epithelial cells to form in spheroids. This mechanism would be like the thickening of the intestinal mucosa due to the accumulation of additional layers of epithelial cells when the Wnt/β-catenin pathway is mutationally activated^37^. Furthermore, RPPA profiling unveiled only a subtle reduction in BCL2L1/Bim expression in MCF10A overexpressing *CEACAM5*. This finding aligns with the previous observation indicating that the formation of MCF10A spheroids with hollowed out interiors relies on Bim's role in initiating apoptosis^38^.

In conclusion, our findings suggest that expanding concurrent screening for drivers and dependencies to encompass thousands of genes could unveil novel therapeutic targets. However, the execution of such large-scale screening surpasses the capabilities of individual laboratories. Furthermore, our study highlights the efficacy of proteomic profiling using RPPA in elucidating the biochemical mechanisms underlying oncogenic phenotypes.

## Methods

### Barcoded-ORF library and shRNA library generation

The barcoded-ORF library was constructed by Dr. Kenneth Scott’s laboratory at Baylor College of Medicine with the human ORFeome version 8.1 Entry Clone Collection (hORFeome V8) as donors and pLenti6.3/V5 as destination vector using the Gateway recombination-based barcoding pipeline as described^12^. Pooling was done post-lentiviral transduction as described below. The shRNA library pool was designed and constructed at Dr. Michael Hemann’s laboratory at the Koch Institute at MIT with six independently designed shRNA for each gene following published procedure using oligonucleotides bulk synthesized by LC Sciences (LC Sciences OligoMix) that were PCR amplified as described^39^. The amplified shRNAs were then batch cloned into the mir30-retroviral vector LMP and then 20 clones of the library were Sanger sequenced to validate the library.

### Pooled screening

The same set of ORFs were lentivirally transduced into both murine non-malignant NMuMG-Myc mammary epithelial cells and human MCF10A cells. To prepare lentivirus, each individual ORF plasmid was transfected separately along with helper plasmids into 293 T cells using jetPRIME transfection reagent (Polyplus Transfection) and virus was collected after 3 days. MCF10A and NMuMG-Myc cells were individually infected for 24 h, followed by addition of fresh media and 48 h before adding blasticidin to select for stable transfectants. After 8 days the individually transfected cells were then pooled and split into four biological replicates which were cultured for an additional 3 days before dividing them into the four conditions: (1) time zero, from which genomic DNA was isolated and used for PCR amplification of the barcodes; (2) 2D cell culture for 30 days (with periodic splitting to avoid reaching confluence); (3) 3D culture of single cells in matrigel for 30 days; and (4) aliquots comprised of 2 × 10^6^ cells were injected subcutaneously into nude mice for in vivo tumor formation for 8 weeks. Following genomic DNA isolation and PCR amplification of the barcodes, each PCR amplicon product was ligated to a unique index adaptor for construction of a multiplexed Illumina library using the TrueSeq DNA LT Sample Prep Kit (Illumina) for MiSeq sequencing. After mapping reads to the set of utilized barcode sequences, counts for each ORF were normalized by the number of total numbers of mapped reads per amplicon. Following log2 transformation, the four values from the endpoint samples were compared to the corresponding initial values from the zero time point samples as described^12^.

For shRNA screening, the LMP shRNA plasmid library and helper plasmid were transfected into Bing packaging cells with Profection Mammalian Transfection System. After 48 h, the viral supernatant was collected and filtered. Breast cancer cell lines MCF7, Cal120, MDA-MB-453 were infected at a MOI of 0.3 using diluted viral supernatant supplemented with 8 μg/ml polybrene. 72 h after the infection, infected cells were selected using puromycin for 3 days. The cells were then split into separate cultures for generating quadruple replicates for zero time point cultures and quadruple replicates for two-weeks growth in vitro. The cells were collected and genomic DNA was extracted using E.Z.N.A Tissue DNA Kit (Omega Bio-Tek). PCR reactions was done using specific primers: half forward (5′-TAGTGAAGCCACAGATGTA) and bc1-reverse (5′-AAAGCGCATGTCCAGACTGCC) to amplify hairpin sequence. Each PCR amplicon was prepared for Illumina Miseq library using the TrueSeq DNA LT Sample Prep Kit (Illumina), where each amplicon was ligated to unique index Adaptors for MiSeq sequencing. Following normalization and log2 transformation, the quadruple values from the endpoint samples were compared to the corresponding initial values from the zero time point samples as described^39^.

### Animals

One million mammary epithelial cells were trypsinized, resuspended in 100 µl DMEM and injected subcutaneously or orthotopically into mammary pads of four- to five-week-old female nude mice (NCR nu/nu; Envigo Inc., Wilmington, MA). Growth was followed over time by taking caliper measurements at indicated time points. Tumor volume was measured as 0.52 × length × width 2. Prior to tumor excision, mice were euthanized by carbon dioxide asphyxiation. Tumors were excised six-eight weeks post injections or when one of the measurements reached 2 cm.

### Tissue culture and lentiviral infection

MCF10A, T47D, HEK293T, MCF7, MCF10A, NMuMG cells were obtained from the American Type Culture Collection (ATCC). HEK293T and T47D were cultured in DMEM with 10% fetal bovine serum (FBS) and 100 U/ml penicillin streptomycin. MCF10A cells were cultured in DMEM/F12 supplemented with 5% donor horse serum, EGF 20 ng/mL, insulin 10ug/mL, hydrocortisone 100 µg/mL cholera toxin 10 ng/mL and 100 U/ml penicillin streptomycin. MCF7 and NMuMG-myc cells were cultured in DMEM with 10% fetal bovine serum (FBS), insulin 10ug/mL and 100 U/mL penicillin streptomycin. To produce lentiviral particles, HEK293T cells were transiently transfected with transfer, envelope, and packaging plasmids using Profection Mammalian Transfection System (Promega). The viral supernatant was collected 24 h later. For lentiviral infection, MCF10A cells were grown in a 1:4 mixture of the growth medium and the viral supernatant for 24 h. Two days post infection, blasticidin (Invivogen) selection was performed for 8 days to select for positive clones and these cells were used for various assays. Colony formation assays were performed by plating 1000 cells in triplicate on 6-well plates. Medium was changed every 3 days. After 3 weeks culture, cells were methanol fixed and stained with 0.5% crystal violet. After pictures were taken, crystal violet was dissolved with 0.1% SDS overnight. Dissolved crystal violet staining was read at 595 nm using the Victor 3 machine (Perkin Elmer). 3D culture was performed as previously reported^40^ using growth factor-reduced matrigel (BD Bioscience).

### RPPA and immunoblotting

RPPA was performed by the MD Anderson RPPA Core Facility using protein lysates. For each RPPA replicate, cells were washed with cold PBS twice and lysed with 100 μL lysis buffer (1% Triton X-100, 50 mM HEPES, pH 7.4, 150 mM NaCl, 1.5 mM MgCl2, 1 mM EGTA, 100 mM NaF, 10 mM Na pyrophosphate, 1 mM Na3VO4 and 10% glycerol, containing freshly added protease and phosphatase inhibitors from Roche Applied Science Cat. # 05056489001 and 04906837001, respectively), and mixed with 4 × SDS sample buffer (40% Glycerol, 8% SDS, 0.25 M Tris–HCl, pH 6.8). Immunoblotting was carried out using antibodies against Ceacam5 (Sigma), Akt, phospho AKT (Cell Signaling and Actin (Abcam) or GAPDH (Santa Cruz) as loading control. Cells were lysed in lysis buffer (25 mM Tris, pH 7, 150 mM NaCl, 0.1% SDS, 0.5% sodium deoxycholate, 1% Triton X 100) and cocktail protease inhibitors (Roche) and centrifuged to clear the cell lysates. The cell lysates were then loaded on a 10% SDS-PAGE, and the proteins were subsequently transferred onto a nitrocelluose membrane. The membrane was blocked with 5% milk in TBST (20 mM Tris–HCl, pH7.5, 150 mM NaCl, 0.1% Tween-20) for 30 min and then incubated with antibodies in 5% milk in TBST overnight. After three extensive washes for 10 min each in TBST, the membrane was incubated with the secondary antibody, horseradish peroxidase (HRP)-conjugated anti-rabbit (Santa Cruz) or anti-mouse IgG antibody (Abcam) for 1 h, followed by three washes in TBST. The membrane was developed with the chemiluminescent kit (Biorad) and exposed to X-ray films (Kodak).

### Other methods

For real-time RT-PCR, total mRNA was isolated from the indicated cells using the RNeasy Mini Kit (Qiagen). cDNAs were synthesized using the cDNA Synthesis Kit (Thermo Fisher Scientific) according to the manufacturer’s instructions. The transcript level of GAPDH was used as an internal control. The amplification steps consisted of 10 min at 50 °C and 5 min at 95 °C, followed by 40 cycle of denaturation for 10 s at 95 °C and annealing/extension for 30 s at 60 °C. All samples were analyzed in triplicate, and the relative gene expression was calculated according to the comparative threshold cycle (ΔΔCt) method.

Student’s t-test was used to determine the significance of difference of means measurements, using the standard p < 0.05 cutoff. Error bars represent the standard deviation of data.

For analysis of 3D Matrigel cultures, spheroids were photographed and their diameters were assessed using Leica Qwin Standard V2.6 software (https://www.leica-microsystems.com/). For immunofluorescence staining, acini were fixed in 4% paraformaldehyde, washed in PBS, and embedded in optimum cutting temperature (OCT) compound (Tissue-Tek) and then processed by regular histological procedures. 4′, 6-diamidino-2-phenylindole (DAPI) (Molecular Probes) was used as DNA stain.

### Supplementary Information

Supplementary Figure S1.

Supplementary Legends.

Supplementary Table S1.

Supplementary Table S2.

## Data Availability

The datasets used and/or analysed during the current study available from the corresponding author on reasonable request.
