# Supplementary material for: Simultaneous screening of overexpressed genes in breast cancer for oncogenic drivers and tumor dependencies
Source: Sci Rep. 2024 Jun 9;14:13227. doi: 10.1038/s41598-024-64297-w (PMC11162420; doi:10.1038/s41598-024-64297-w)
Supplement: Supplementary file 1 — Supplementary Figure S1. [file 41598_2024_64297_MOESM1_ESM.docx]

Supplementary Figure S1

**Figure S1**. Expression of *CEACAM5* in transfectants of MCF10A and NMuMG and effects on tumorigenicity. a) quantification of real-time RT-PCR measuring expression of *CEACAM5* relative to that of GAPDH in NMuMG transfectants. b) one million cells of NMuMG-myc or NMuMG-myc-stably expressing CEACAM5 were trypsinized and resuspended in 100uL DMEM and injected into the mammary pads of five to six week old female nude mice. Growth was followed over time by taking caliper measurements at the indicated time points. c) comparison of final tumor volume of NMuMG *CEACAM5* transfectant to control; d) quantification of real-time RT-PCR measuring expression of *CEACAM5* relative to that of GAPDH in MCF10A transfectants. The significance of the findings was assessed using a Student's t-test, while error bars depict the standard error of the means.
