# Supplementary material for: Simultaneous screening of overexpressed genes in breast cancer for oncogenic drivers and tumor dependencies
Source: Sci Rep. 2024 Jun 9;14:13227. doi: 10.1038/s41598-024-64297-w (PMC11162420; doi:10.1038/s41598-024-64297-w)
Supplement: Supplementary file 2 — Supplementary Legends. [file 41598_2024_64297_MOESM2_ESM.docx]

Supplementary Table Legends

Supplementary Table 1. Characterization of the overexpression of the 100 genes chosen for this study. Cancer vs. normal differential analysis of breast cancer TCGA data was performed with Oncomine Research tools (Invasive Ductal vs. Normal; Invasive Lobular vs. Normal) as was outlier analysis (COPA 90; COPA 95).

Supplementary Table 2. Values and statistical tests of log2-fold changes in the seventy-two ORF proportions for the three growth and/or tumor assays.
